# Optimized Breath-Hold Compressed-Sensing 3D MR Cholangiopancreatography at 3T: Image Quality Analysis and Clinical Feasibility Assessment

**DOI:** 10.3390/diagnostics10060376

**Published:** 2020-06-05

**Authors:** Ji Soo Song, Seung Hun Kim, Bernd Kuehn, Mun Young Paek

**Affiliations:** 1 Department of Radiology, Jeonbuk National University Medical School and Hospital, Jeonju 54907, Korea; 2 Research Institute of Clinical Medicine of Jeonbuk National University, Jeonju 54907, Korea; 3 Biomedical Research Institute of Jeonbuk National University Hospital, Jeonju 54907, Korea; 4 Department of Internal Medicine, Jeonbuk National University Medical School, Jeonju 54907, Korea; shkimgi@jbnu.ac.kr; 5 Siemens Healthineers Ltd., 91052 Erlangen, Germany; bernd.kuehn@siemens-healthineers.com; 6 Siemens Healthineers Ltd., Seoul 03737, Korea; munyoung.paek@siemens-healthineers.com

**Keywords:** data compression, magnetic resonance imaging, breath-holding, cholangiopancreatography, magnetic resonance, imaging, three-dimensional

## Abstract

Magnetic resonance cholangiopancreatography (MRCP) has been widely used in clinical practice, and recently developed compressed-sensing accelerated MRCP (CS-MRCP) has shown great potential in shortening the acquisition time. The purpose of this prospective study was to evaluate the clinical feasibility and image quality of optimized breath-hold CS-MRCP (BH-CS-MRCP) and conventional navigator-triggered MRCP. Data from 124 consecutive patients with suspected pancreaticobiliary diseases were analyzed by two radiologists using a five-point Likert-type scale. Communication between a cyst and the pancreatic duct (PD) was analyzed. Signal-to-noise ratio (SNR) of the common bile duct (CBD), contrast ratio between the CBD and periductal tissue, and contrast-to-noise ratio (CNR) of the CBD and liver were measured. Optimized BH-CS-MRCP showed significantly fewer artifacts with better background suppression and overall image quality. Optimized BH-CS-MRCP demonstrated communication between a cyst and the PD better than conventional MRCP (96.7% vs. 76.7%, *p* = 0.048). SNR, contrast ratio, and CNR were significantly higher with optimized BH-CS-MRCP (*p* < 0.001). Optimized BH-CS-MRCP showed comparable or even better image quality than conventional MRCP, with improved visualization of communication between a cyst and the PD.

## 1. Introduction

Magnetic resonance cholangiopancreatography (MRCP) visualizes not only anatomic variations of the biliary system but also various pathologies such as biliary stone disease, inflammation, and malignancy [1,2,3,4]. In the past few decades, MRCP has been widely used in clinical practice, and has evolved from two-dimensional (2D) thick-slab MRCP to three-dimensional (3D) MRCP [5,6,7]. Conventional 3D isotropic MRCP (conventional MRCP) is advantageous compared with 2D thick-slab MRCP because it allows for views in multiple, nonorthogonal projections. However, it has some drawbacks, such as long acquisition time, due to the use of a navigator-triggering (NT) technique, and altered image qualities, due to severe motion artifacts in up to 22.2% of patients [8]. The exact reason for altered images in conventional MRCP is not known, but it may be related to the need for an inadequately long acquisition time, especially in patients with an irregular breathing pattern or irritability [7,9,10].

Recently developed compressed-sensing accelerated MRCP (CS-MRCP) has shown great potential in shortening the acquisition time, and has made single breath-hold CS-MRCP (BH-CS-MRCP) possible in clinical practice [8,11,12,13]. However, previous studies have noted some problems with BH-CS-MRCP, such as inferior background signal suppression and lesser visibility of small ductal structures, including the pancreatic duct (PD) and the peripheral intrahepatic bile ducts (IHD) [8,13]. In order to solve these problems, an optimized BH-CS-MRCP with a smaller field-of-view (FOV) and decreased acceleration factor was developed. Although a recent study on modifications of BH-CS-MRCP that used a smaller field-of-view (FOV) with higher spatial resolution and saturation bands showed that modified BH-CS-MRCP was better than original BH-CS-MRCP and was comparable to NT-CS-MRCP in both image quality and detecting PD abnormalities [14], previous studies have been limited to a small number of patients or volunteers, and only focused on qualitative image analysis comparing conventional MRCP and CS-MRCP (whether using the respiratory triggered method or the breath-hold method), or CS-MRCP itself. To our knowledge, this is the first study with a direct comparison of conventional MRCP and optimized BH-CS-MRCP with a large cohort using quantitative image analysis. The purpose of this study was to compare the images acquired with conventional MRCP and optimized BH-CS-MRCP with a smaller FOV and decreased acceleration factor, both qualitatively and quantitatively, in a large number of consecutive patients.

## 2. Materials and Methods

### 2.1. Patients

This prospective study was approved by our site’s institutional review board (IRB File No. 2017-05-019; approved date, 12 June 2017). Written informed consent was obtained from all patients prior to participation in the study. From July 2017 to November 2017, a total of 126 consecutive patients were recruited and underwent conventional NT 3D-MRCP using sampling perfection with application-optimized contrasts (SPACE) and breath-hold 3D CS-MRCP (CS SPACE; Siemens Healthineers) using SPACE and high undersampling combined with CS reconstruction on a 3T scanner. Two postoperative follow-up patients (bile duct resection state) were excluded. Our final sample included a total of 124 patients (58 men and 66 women; mean age, 64.5 years; age range, 18–86 years). Indications for the MRCP were as follows: biliary or pancreatic ductal dilatation (*n* = 42), branch-duct type intraductal papillary mucinous neoplasm (BD-IPMN, *n* = 30), cystic pancreatic lesions other than BD-IPMN (*n* = 9), biliary or pancreatic malignancy (*n* = 9), cholecystectomy or adenomyomatosis (*n* = 8), pancreatitis (*n* = 6), no abnormality in pancreaticobiliary system (*n* = 8), and other (*n* = 12). The reference standard for the diagnosis of pancreaticobiliary pathology was based on subsequent ERCP, EUS, surgery, or follow-up images.

### 2.2. MR Imaging Techniques

All MR examinations were performed on a 3T MR scanner (Magnetom Skyra, Siemens Healthineers, Erlangen, Germany) using an 18-channel body matrix coil combined with a 32-channel spine matrix coil. Patients fasted for at least 4 h before the examination. No spasmolytic drug or negative oral contrast was used. The order of acquisition of conventional MRCP and optimized BH-CS-MRCP was randomized.

#### 2.2.1. Conventional MRCP

Magnetic resonance cholangiopancreatography was performed using 3D SPACE in combination with reduced volume excitation and the NT prospective acquisition correction technique [7,15]. Detailed acquisition parameters are as follows: field-of-view (FOV), 380 × 380 mm^2^; repetition time (TR), variable depending on the respiratory rate; echo time (TE), 519 ms; flip angle (FA), 100°; acceleration factor, 3; number of excitations (NEX), 1.4; spectrally selective fat saturation to suppress signal intensity from fat; matrix, 380 × 380; section thickness, 1 mm; resolution (interpolated), 1 × 1 × 1 mm (0.5 × 0.5 × 1 mm); number of coronal sections, 72; and acquisition time varying with the breathing pattern of the patient.

#### 2.2.2. Compressed Sensing MRCP

Compressed sensing MRCP was performed using a prototypical 3D SPACE sequence with an incoherent undersampling scheme and CS reconstruction technique (CS SPACE, Siemens Healthineers, Erlangen, Germany). In this prototype sequence, incoherent undersampling was obtained using a Poisson-Disk pattern in 2 phase-encoding dimensions [11]. In addition, the fluctuation of echo train trajectories due to irregular sampling of k-space was mitigated by increasing the smoothness of train trajectories [16]. For optimized BH-CS-MRCP, an acceleration factor of 17 (5.7% k-space data sampling) was used. Detailed acquisition parameters were as follows: FOV, 384 × 192 mm^2^; TR/TE, 1700/503 ms; FA, 110°; spectrally selective fat saturation to saturate fat signal intensity; NEX, 1; section thickness, 1 mm; resolution (interpolated), 1 × 1 × 1 mm (0.5 × 0.5 × 1 mm); and number of coronal sections, 72. Acquisition time was 16 s. The CS reconstruction technique used a regularization parameter of 0.003 and 14 iterations. The inline image reconstruction took approximately 4–5 min for each data set.

### 2.3. Image Analysis

#### 2.3.1. Qualitative Image Analysis

Two radiologists (with 10 and 2 years of experience in abdominal MR imaging) independently reviewed the conventional MRCP and optimized BH-CS-MRCP examinations within a 4-week interval in order to minimize recall bias. All of the images were anonymized and distributed to the reviewers in a random order, and the radiologists were blinded to acquisition methods during image analysis. A two-dimensional thick-slab MRCP was used as a reference standard. The radiologists were able to adjust the window level and width during the image analysis. No data from other sequences of the MR examination were made available to the readers.

A 5-point Likert-type scale was used to grade image parameters. These included the degree of image quality degradation by artifacts, background suppression, and overall image quality (Table 1).

The reviewers also evaluated the visualization of 9 segments of the pancreaticobiliary system: the common bile duct (CBD); bilateral first and second intrahepatic ducts (IHDs); cystic duct insertion; and pancreatic duct (PD) in the proximal, middle, and distal segments. For each segment, ductal visualization was also graded on a 5-point Likert-type (Table 1). An average score from the 2 reviewers was used to determine adequate visualization of the ductal system. Adequate visualization of the entire biliary system was determined if the CBD, Right IHD (Rt IHD), Left IHD (Lt IHD), and cystic duct visualization all achieved an average score of 3 or higher. Similarly, adequate visualization of the entire PD was determined if the proximal, middle, and distal pancreatic duct all achieved an average score of 3 or higher. The presence of communication between a cystic lesion and the PD was assessed by analyzing the 3D images as well as source images of conventional MRCP and optimized BH-CS-MRCP.

#### 2.3.2. Quantitative Image Analysis

**Figure 1:**
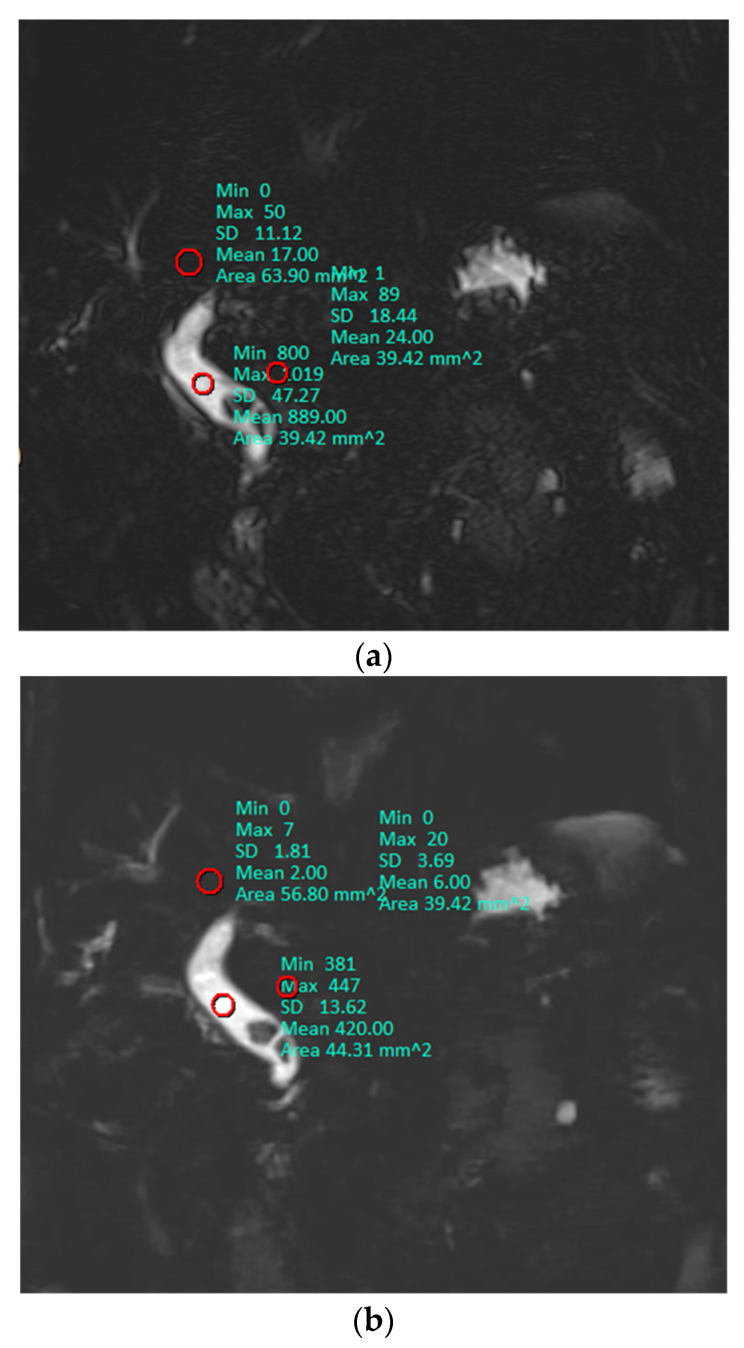
A representative slice used in each patient for quantitative analysis. The mean value of ROI (red circles) represents signal intensity (SI) and standard deviation (SD) was used as image noise. Signal-to-noise ratio, contrast ratio, and contrast-to-noise ratio were 18.81, 0.95, and 25.40 on conventional MRCP (**a**), and 30.84, 0.97, and 43.02 on optimized BH-CS-MRCP (**b**), respectively.

Research personnel with 1 year of experience performed quantitative image analysis of the source images. Similar to the previous reports, 1 representative slice level that depicted the center of the common bile duct (CBD) in each patient was selected, and the signal intensity (SI) of the CBD and periductal tissues was measured by applying regions of interest (ROIs) [17,18]. ROIs for the SI of the CBD were placed in homogeneous, artifact-free areas of the CBD in the middle third of its course. ROIs for the SI of periductal tissue and the liver were placed in homogeneous, artifact-free areas adjacent to the SI of CBD. Image noise was defined as the standard deviation (SD) of the CBD, periductal tissue, and the liver from the same ROIs as that for SI because the background noise was too low (Figure 1) [19]. The signal-to-noise ratio (SNR) of the CBD and contrast ratio between the CBD and periductal tissues on 3D MRCP was evaluated quantitatively using the following formulas:

SNR = SI_CBD_/SD_CBD_ (1)

Contrast ratio = (SI_CBD_ − SI_periductal tissue_)/(SI_CBD_ + SI_periductal tissue_) (2)

According to previous reports [18], we calculated the contrast-to-noise ratio (CNR) between the CBD and the liver using the following formula:

CNR = (SI_CBD_ − SI_liver_)/{[(SD_CBD_)^2^ + (SD_liver_)^2^]/2}^1/2^ (3)

### 2.4. Statistical Analysis

All numerical values are reported as mean ± standard deviation. The Wilcoxon signed-rank test was used to compare the qualitative scores. A paired *t*-test was used to compare SNR, contrast ratio, and CNR. Fisher’s exact test was used to compare the presence of communication between a cyst and the PD. Interobserver agreement was determined using Cohen’s kappa coefficients. Kappa coefficients were interpreted as follows: poor agreement, <0.20; fair, 0.20–0.39; moderate, 0.40–0.59; substantial, 0.60–0.79; and almost perfect, ≥ 0.80. McNemar’s test was used to compare the number of patients with non-diagnostic or poor image quality (an average overall qualitative score of ≤ 2) for the biliary system and the PD.

All statistical analyses were performed using MedCalc version 18.6 (MedCalc Software, Ostend, Belgium), and *p* < 0.05 was considered statistically significant.

## 3. Results

### 3.1. Interobserver Agreement

Interobserver agreement of qualitative analysis was moderate to almost perfect for conventional MRCP (κ = 0.59–0.80) and moderate to substantial for optimized BH-CS-MRCP (κ = 0.57–0.79).

### 3.2. Qualitative Analysis

**Figure 2:**
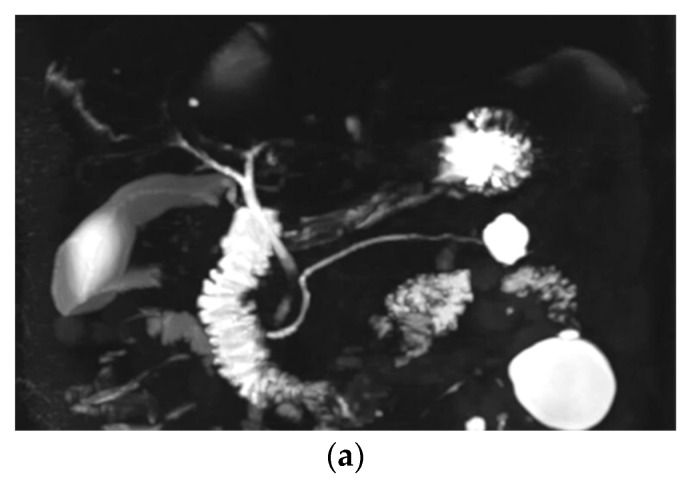

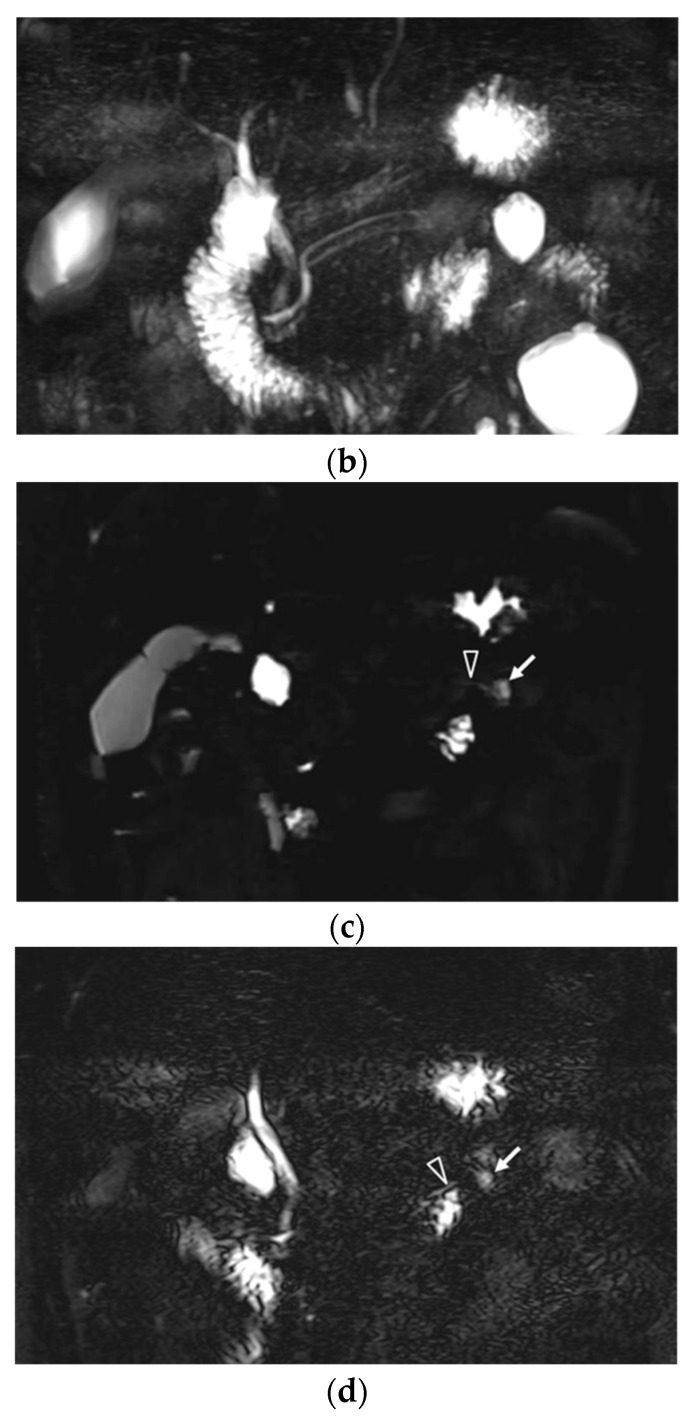
A 59-year-old male with a 2.4 cm sized branch-duct type intraductal papillary mucinous neoplasm (BD-IPMN) in the tail portion. Overall image quality (2 vs. 4, conventional MRCP vs. optimized BH-CS-MRCP, respectively), image degradation by artifacts (3 vs. 5), and background suppression (3 vs. 4) were all better with optimized BH-CS-MRCP (**a**) than those with conventional MRCP (**b**). BD-IPMN (arrow) is more clearly visualized on optimized BH-CS-MRCP, and the presence of communication with pancreatic duct (hollow arrowhead) was well visualized on the source image of optimized BH-CS-MRCP (**c**), while on the source image of conventional MRCP (**d**), the communication could not be seen.

Image quality degradation by artifacts, background suppression, overall image quality, duct visualization of the CBD, Lt first level IHD, cystic duct, middle and distal PD, and communication between a cyst and the PD were significantly higher with optimized BH-CS-MRCP than those with conventional MRCP (*p* < 0.05, Table 2). However, duct visualization of the Rt first level IHD (conventional MRCP vs. optimized BH-CS-MRCP, 4.18 ± 1.16 vs. 4.38 ± 1.02, *p* = 0.079), Rt (3.54 ± 1.29 vs. 3.40 ± 1.34, *p* = 0.180) and Lt second level IHD (3.60 ± 1.15 vs. 3.81 ± 1.12, *p* = 0.096), and proximal PD (4.28 ± 0.98 vs. 4.44 ± 0.93, *p* = 0.087) did not show a significant difference between conventional MRCP and optimized BH-CS-MRCP. There were 30 patients with BD-IPMN who were considered to have communication between a cyst and the PD, while 23 patients (76.7%) on conventional MRCP and 29 patients (96.7%) on optimized BH-CS-MRCP demonstrated the presence of such communication (*p* = 0.048, Figure 2).

### 3.3. Quantitative Analysis

The SNR of the CBD with optimized BH-CS-MRCP was 40.8% higher than that with conventional MRCP (*p* < 0.001). The contrast ratio between the CBD and periductal tissue with optimized BH-CS-MRCP was 5.3% higher than that with conventional MRCP, and CNR between the CBD and the liver with optimized BH-CS-MRCP was 52.3% higher than that with conventional MRCP (*p* < 0.001, Table 3).

### 3.4. Patients with Non-Diagnostic or Poor Image Quality

**Figure 3:**
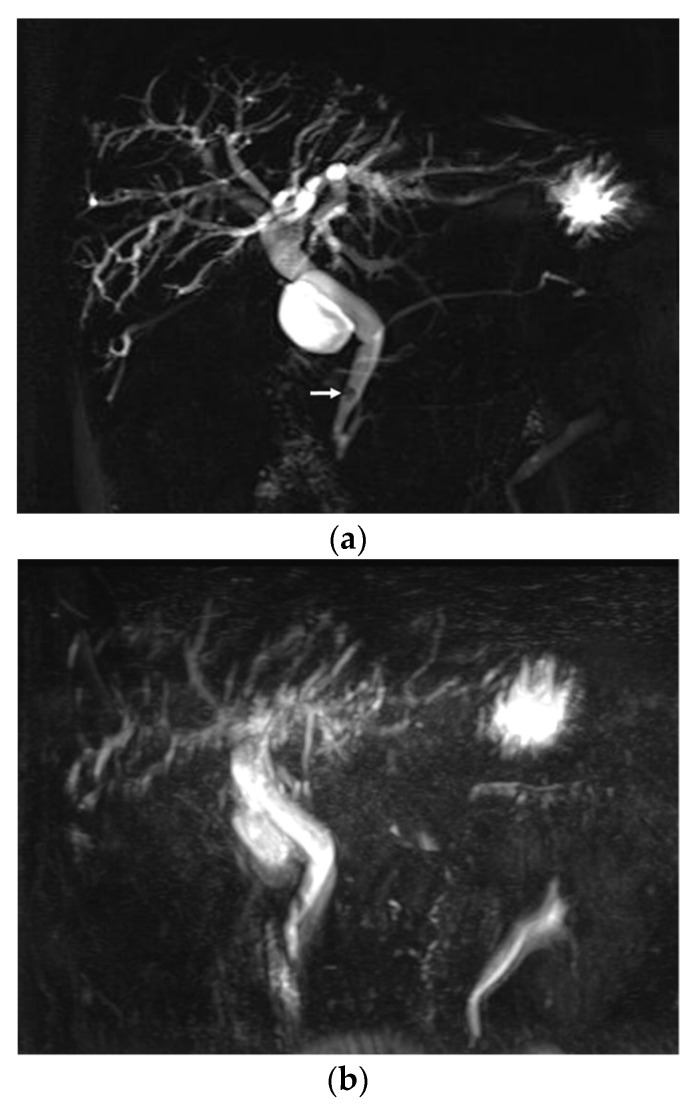

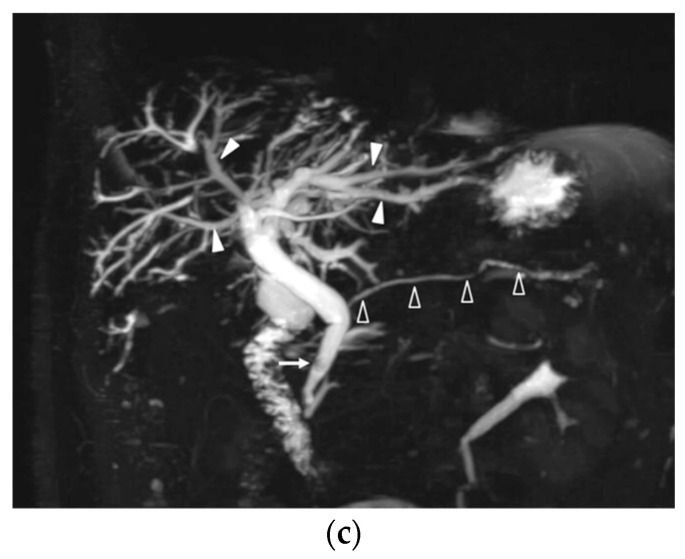
A 61-year-old male with a 5 mm sized distal CBD stone. A 2D thick-slab MRCP (**a**), as a reference standard, clearly depicts distal CBD stone (arrow) with upstream biliary dilatation. On conventional MRCP (**b**), the overall image quality is poor (score 1) mainly due to image degradation by severe artifacts (score 2), and the evaluation for the presence of distal CBD stone is limited. On optimized BH-CS-MRCP (**c**), overall image quality, image degradation by artifacts, and background suppression were excellent (score 5). In addition, visualization of distal CBD stone (arrow) as well as second branch IHD (solid arrowheads) and pancreatic duct (hollow arrowheads) is seen (score 5 for all of the pancreaticobiliary system).

**Figure 4:**
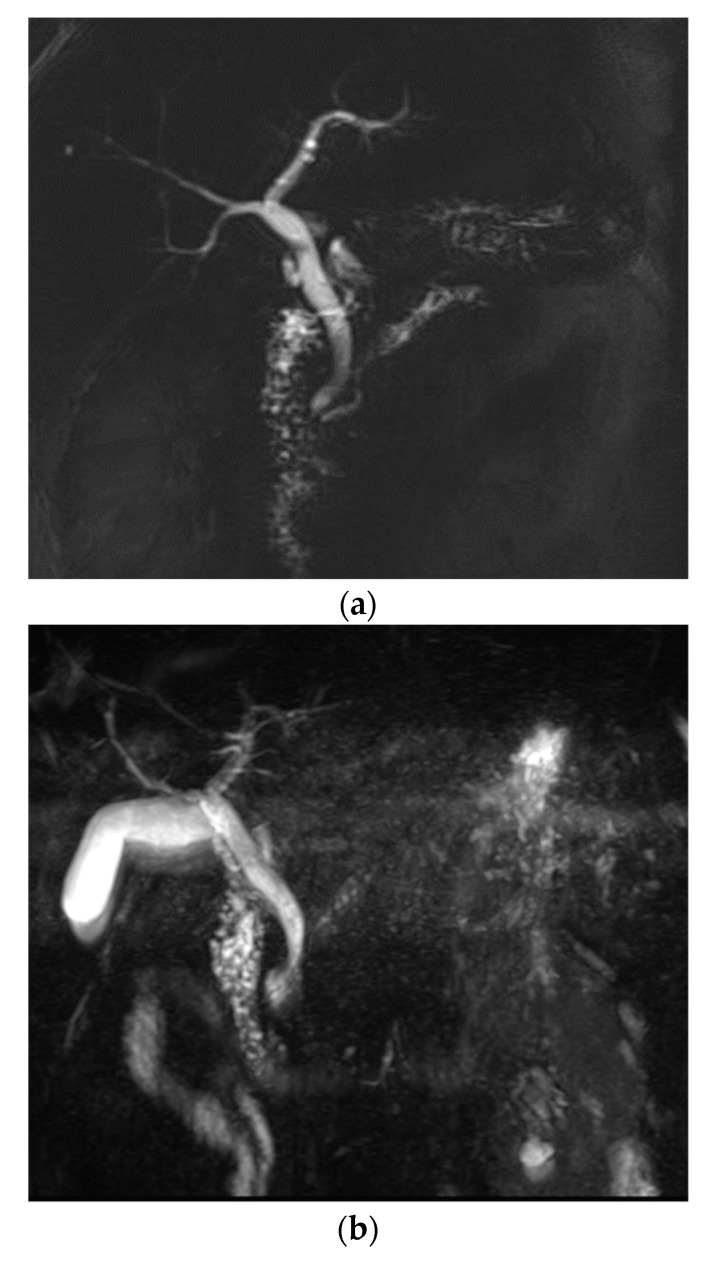

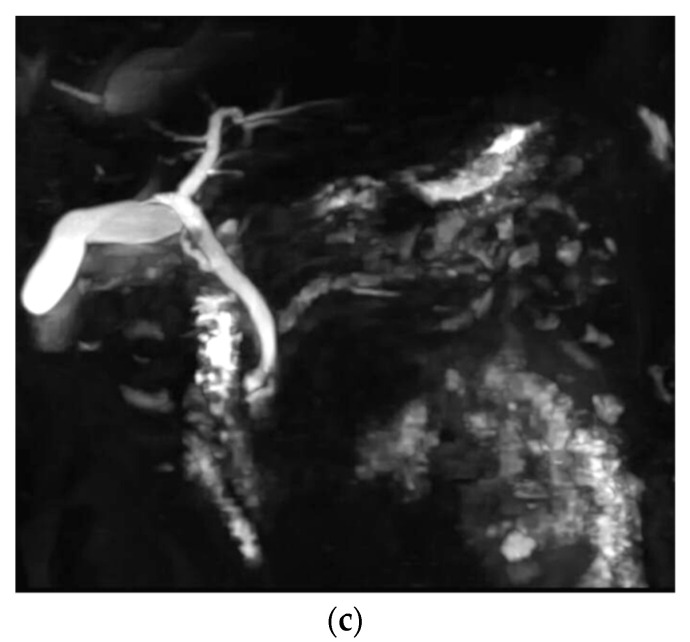
A 57-year-old male with chronic pancreatitis. A 2D thick-slab MRCP (**a**), as a reference standard, clearly depicts dilated pancreatic duct with multiple filling defects, compatible with chronic pancreatitis. Overall image quality was comparable between conventional MRCP (**b**) (score 3) and optimized BH-CS-MRCP (**c**) (score 3). However, image degradation by artifacts (3 vs. 4.5) and background suppression (3 vs. 3.5) was better with optimized BH-CS-MRCP. The pancreatic duct is well depicted on optimized BH-CS-MRCP, while it is hardly recognized on conventional MRCP. In addition, the visibility of CBD and IHD was all better with optimized BH-CS-MRCP, except for the Rt 2nd level IHD (4 vs. 1).

On conventional MRCP, 27 patients (21.8%) were considered to have non-diagnostic or poor image quality based on the overall image quality score (Figure 3). In contrast, 13 patients (10.5%) were considered to have non-diagnostic or poor image quality on optimized BH-CS-MRCP (*p* = 0.014). Of patients with poor image quality on conventional MRCP, 21 patients had an image quality score of ≥3 on optimized BH-CS-MRCP. Six patients (4.8%) had poor image quality on both conventional and optimized BH-CS-MRCP. One hundred patients (80.6%) with conventional MRCP and 113 patients (91.1%) with optimized BH-CS-MRCP were considered to have adequate visualization of the PD (*p* = 0.011, Figure 4).

## 4. Discussion

In this study, conventional 3D MRCP and optimized BH-CS-MRCP were performed on a large number of consecutive patients suspected of having various pancreaticobiliary diseases. Based on both qualitative and quantitative analysis results, optimized BH-CS-MRCP was considered to be better than conventional MRCP. Most ductal structures were graded significantly higher on optimized BH-CS-MRCP; the detection of communication between a cyst and the PD was better visualized on optimized BH-CS-MRCP as well.

Several recent studies compared the image quality of the BH-CS-MRCP to conventional MRCP. One of the earliest studies on CS-MRCP by Chandarana et al. comparing conventional MRCP and BH-CS-MRCP demonstrated similar or superior image quality for the CBD and PD visualization; however, this difference was not statistically significant [11]. Zhu et al. compared conventional MRCP, NT-CS-MRCP, and BH-CS-MRCP and found that although BH-CS-MRCP depicted bile ducts clearly with high efficiency, visualizing small ductal structures such as the peripheral IHD, PD, and communication between a cystic lesion and the PD was limited, with adequate PD visualization seen in 90%, 82.5%, and 76.3% of patients, respectively. In addition, conventional MRCP and BH-CS-MRCP demonstrated no significant difference in the diagnosis of communication between a cyst and the PD, while NT-CS-MRCP had a superior diagnostic capacity compared to the other two modalities [13]. Another study showed that the adequate visualization of the PD was achieved in 90.7% with NT-CS-MRCP, 78.7% with original BH-CS-MRCP, and 89.3% with modified BH-CS-MRCP [14]. In the current study, we found adequate PD visualization with communication between a cyst and the PD significantly better demonstrated with optimized BH-CS-MRCP as compared to conventional MRCP.

The major difference between a previous study evaluating modified BH-CS-MRCP and our study is that we only reduced the FOV and matrix without changes in spatial resolution, used an acceleration factor of 17 (5.7% k-space data sampling) instead of 28 (3.6% k-space data sampling), and used a regularization factor with iterations of 0.003 instead of 0.002 and 14 instead of 20 [14]. Interestingly, previous studies showing inferior visualization of small branches of the IHD or the PD in evaluations of conventional MRCP, NT-CS-MRCP, or BH-CS-MRCP used acceleration factors of 24 or 28, and 20 iterations [13,20]. In order to improve the image quality of BH-CS-MRCP in our hospital, we tested various acquisition parameters in preliminary studies including acceleration factor, regularization factor, and the number of iterations. Although those data are not shown and not compared to data presented in this paper, we assume that decreasing an acceleration factor in order to achieve more k-space data and a fewer number of iterations may have resulted in the overall better image quality of BH-CS-MRCP achieved in our study as compared to prior research. In addition, the acquisition time of 16 s could still be achieved due to reduced number of slices acquired (104 vs. 72) and reduced NEX (1.4 vs. 1), although we used lower acceleration factor (28 vs. 17) compared with modified BH-CS-MRCP by Zhu et al. [14]. Furthermore, since BH-CS-MRCP is a ‘breath-hold’ examination, the image quality is largely dependent on the patient’s compliance as well as the capability to breath-hold. Our MR radiographers are well trained in improving patient compliance, through the provision of good instructions in preparation for breath-holding image acquisition, as previously described [21,22].

Although a 16 s breath-holding sequence is considered achievable in most cases, some patients may have barriers that limit compliance (e.g., impaired mental status, dyspnea, or hearing loss). A previous study demonstrated better duct visibility and higher diagnostic performance for detecting malignancy with conventional MRCP in noncooperative patients, compared to BH-MRCP [10]. NT MRCP thus may be indicated for noncooperative patients. In addition, many patients referred for MRCP could have various comorbidities that could reduce tolerance for long examination times. In these cases, optimized BH-CS-MRCP might lead to superior image quality compared to conventional MRCP.

In the current study, the SNR, contrast ratio, and CNR obtained using optimized BH-CS-MRCP were significantly higher than those obtained with conventional MRCP. Although the SI of the CBD, liver, and periductal tissue were lower on optimized BH-CS-MRCP compared to those on conventional MRCP, the dramatic decrease in image noise resulted in a better ratio of quantitative analysis. In a previous study by Yoshida et al., the gradient and spin-echo (GRASE) sequence with single breath-hold significantly improved the CNR of the CBD compared with conventional MRCP, with a 95% shorter acquisition time [23]. Although most of the qualitative scores were significantly better with GRASE, no significant differences in qualitative scores were found between GRASE and conventional MRCP for anterior, posterior, and segment three branches. Our study results were similar, with Rt first level, Rt and Lt second level IHD, and proximal PD showing no significant difference between conventional MRCP and optimized BH-CS-MRCP. Since most cases showed equivalent image quality of small ductal structures, optimized BH-CS-MRCP should be the initial diagnostic modality of choice, whereas conventional MRCP should only be used in cases in which poor image quality (especially small ductal structures) was obtained using optimized BH-CS-MRCP.

Our study has several limitations. First, this was a single-center prospective study, and therefore this may limit generalizability; Second, the reference standards used for measurement in our study were based on a combination of ERCP, EUS, surgery, and follow-up imaging findings; Third, since the calculation of parameters such as SNR and CNR in the context of parallel imaging or compressed-sensing technique is technically difficult and usually not feasible clinically, the result of our study should be carefully interpreted [24]; Finally, we lacked comparison images from NT-CS-MRCP. Future studies comparing conventional MRCP, optimized BH-CS-MRCP, and NT-CS-MRCP are needed.

In conclusion, optimized BH-CS-MRCP provided comparable or even better image quality than conventional MRCP, with superior visualization of communication between a pancreatic cyst and the PD. We recommend using an optimized BH-CS-MRCP as a first-line 3D MRCP, and in patients with poor breath-holding capability, images from conventional MRCP should be acquired.

## Figures and Tables

**Table 1:** Image quality parameter scores.

Parameter	Scoring System
Image quality degradation by artifacts	1 = nondiagnostic image due to severe artifacts 2 = major artifacts causing significant problem in diagnosis 3 = moderate artifacts with some uncertainty in diagnosis 4 = minor artifacts without problems in diagnosis 5 = excellent image quality without any detectable artifacts
Background suppression	1 = significant background signal that rendered image interpretation impossible 2 = remarkable background signal that rendered image interpretation difficult 3 = noticeable background signal that is distracting in image interpretation 4 = minimal background signal without problems in observation of pancreaticobiliary tree 5 = excellent background suppression
Overall image quality	1 = nondiagnostic image 2 = below average image quality 3 = average image quality 4 = good image quality 5 = excellent image quality
Ductal visualization	1 = ductal structure not visible 2 = ductal structure vaguely identified 3 = ductal structure partially visible 4 = most of the ductal structure visible with some blurring 5 = entire ductal structure visible with excellent details

**Table 2:** Comparison of image quality between conventional MRCP and BH-CS-MRCP.

	Conventional MRCP	Optimized BH-CS-MRCP	*p* Value
Image quality degradation by artifacts	4.01 ± 1.03 (1–5)	4.49 ± 0.72 (2–5)	< 0.001
Background suppression	3.65 ± 0.64 (2–5)	3.99 ± 0.64 (2–5)	< 0.001
Overall image quality	3.58 ± 1.21 (1–5)	3.90 ± 0.96 (2–5)	0.009
Duct visualization			
CBD	4.39 ± 0.82 (2–5)	4.69 ± 0.60 (3–5)	0.001
Rt 1^st^ level IHD	4.18 ± 1.16 (1–5)	4.38 ± 1.02 (1–5)	0.079
Lt 1^st^ level IHD	4.38 ± 0.92 (1–5)	4.68 ± 0.64 (1–5)	0.001
Rt 2^nd^ level IHD	3.54 ± 1.29 (1–5)	3.40 ± 1.34 (1–5)	0.180
Lt 2^nd^ level IHD	3.60 ± 1.15 (1–5)	3.81 ± 1.12 (1–5)	0.096
Cystic duct	3.30 ± 1.36 (1–5)	3.70 ± 1.16 (1–5)	0.001
Proximal PD	4.28 ± 0.98 (1–5)	4.44 ± 0.93 (1–5)	0.087
Middle PD	4.06 ± 1.28 (1–5)	4.39 ± 0.96 (1–5)	0.010
Distal PD	3.98 ± 1.32 (1–5)	4.25 ± 0.99 (1–5)	0.038
Communication between PD and cyst	76.7% (23/30)	96.7% (29/30)	0.048

Note. Values are mean ± standard deviation (range). MRCP = magnetic resonance cholangiopancreatography, BH-CS-MRCP = breath-hold compressed-sensing-accelerated magnetic resonance cholangiopancreatography, CBD = common bile duct, IHD = intrahepatic bile duct, PD = pancreatic duct.

**Table 3:** Results of quantitative image analysis.

	Conventional MRCP	Optimized BH-CS-MRCP	*p* Value
Signal-to-noise ratio	24.51 ± 14.00	34.52 ± 15.11	< 0.001
Contrast ratio	0.94 ± 0.04	0.99 ± 0.01	< 0.001
Contrast-to-noise ratio	31.51 ± 15.94	48.00 ± 20.89	< 0.001

Note. Values are mean ± standard deviation. MRCP = magnetic resonance cholangiopancreatography, BH-CS-MRCP = breath-hold compressed-sensing-accelerated magnetic resonance cholangiopancreatography.
